# Unravel the Effects of UV Light on the Lattice Stability of Perovskite via Numerical Simulation

**DOI:** 10.1002/advs.202511211

**Published:** 2025-09-24

**Authors:** Qihang Yang, Yuqin Liu, Xi Tao, Yanyan An, Lory Wenjuan Yang, Qin Liu, Ruqiang Dou, Na Liu, Fan Xu, Ryan Taoran Wang, Gu Xu

**Affiliations:** ^1^ Shenzhen Institute for Advanced Study University of Electronic Science and Technology of China Shenzhen 518110 China; ^2^ Materials Science & Engineering McMaster University Hamilton Ontario L8S4L7 Canada; ^3^ Faculty of Materials Science Shenzhen MSU‐BIT University Shenzhen 518115 China

**Keywords:** near space, numerical simulation, perovskite, stability, UV light

## Abstract

The light degradation of perovskite greatly hinders the commercialization of perovskite solar cells, which is yet to be resolved, despite the many attempts. A simulation method is applied here to illustrate the degradation kinetics, which reveals that under dark conditions, these perovskites show minimal degradation, suggesting that water and oxygen have little damaging effect without light. However, exposure to UV light significantly accelerates the degradation, which can be reduced by introducing self‐assembling from Zn^2+^ and 1‐(triazol‐1‐ly)‐4‐(tetrazol‐5‐ylmethyl) benzene (Zn‐TTB) as an additive. This indicates that defect passivation via additives can significantly enhance the durability of perovskite materials. These findings not only provide insights into the kinetics of UV‐induced degradation of perovskites but also highlight the role of additives in improving the longevity of these materials, offering promising directions for their practical application under UV‐exposed conditions.

## Graphical abstract

The perovskite lattice stability against UV light is enhanced via appropriate doping and additive strategies, which promotes their future applications in (near) space environments.

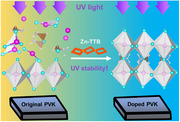

## 1 Introduction

Perovskite photovoltaic materials have attracted significant attention in recent years due to their outstanding optoelectronic properties, low‐cost fabrication, and extensive application scenarios.^[^ ^1^, ^2^, ^3^ ^]^ Hybrid perovskites (such as CH_3_NH_3_PbI_3_, abbreviated as MAPbI_3_) have achieved remarkable breakthroughs in solar cell applications, with power conversion efficiencies (PCE) increasing rapidly from 3.8% to over 27% within a decade.^[^ ^4^ ^]^

However, the intrinsic instability of the perovskite remains a major obstacle to its commercial viability.^[^ ^5^, ^6^ ^]^ The perovskite is not only sensitive to oxygen and moisture, but also to UV irradiation,^[^ ^7^, ^8^ ^]^ resulting in irreversible phase transitions that cease the photovoltaic property.^[^ ^9^, ^10^, ^11^ ^]^ Although the attack from moisture and oxygen could be prevented by the encapsulation, it could not block the UV irradiation. Recent studies have revealed that light‐induced ion migration is detrimental to the perovskite stability.^[^ ^12^ ^]^ Thus, enhancing the structural stability of perovskites under UV irradiation has been the major obstacle.

In order to resolve this challenge, various strategies have been proposed and attempted. For example, some researchers proposed a dual‐interface protection strategy utilizing an ultrathin plasma polymer passivation layer (ultrathin adamantane‐based plasma polymer (ADA) film), which significantly improved device stability.^[^ ^13^, ^14^ ^]^ However, this method could only generate random layer thickness, making it impractical for large‐area devices.^[^ ^14^ ^]^ Others tried to enhance both the PCE and environmental stability of perovskite solar cells through structural optimization to improve light reflection.^[^ ^15^ ^]^ Unfortunately, this technique could only remove UV light from the specific incident angle, thus limiting its practical applications.^[^ ^16^ ^]^ Neither could doping be the answer, due to the phase transition caused by the internal strain from foreign ions.^[^ ^17^, ^18^, ^19^ ^]^ It is evident now that the UV incident light remained a major obstacle after various attempts, due not least to the lack of detailed understanding of the interactions between UV light and perovskite structure, especially the influence of irradiation on the perovskite bonding.

It is therefore the purpose of this work to elucidate the influence of UV light on the microstructure and stability of the perovskites by simulation, as it is next to impossible to achieve a similar goal by experiment. By microscopic statistical modeling, we revealed the stability of the perovskite of various doping concentrations under UV irradiation. The detailed kinetics of the degradation process of various perovskites have been illustrated, which revealed that degradation occurs slowly in dark conditions but accelerates under UV exposure, especially for MAPbI_3_ and MAPbI_1−x_Cl_x_, likely due to the presence of defects. By eliminating these defects with self‐assembling from Zn^2+^ and 1‐(triazol‐1‐ly)‐4‐tetrazol‐5‐ylmethyl) benzene (abbreviated as Zn‐TTB), the degradation rate was significantly reduced, demonstrating the effectiveness of additives in improving stability.

## 2 Numerical Modeling

**Figure 1:**
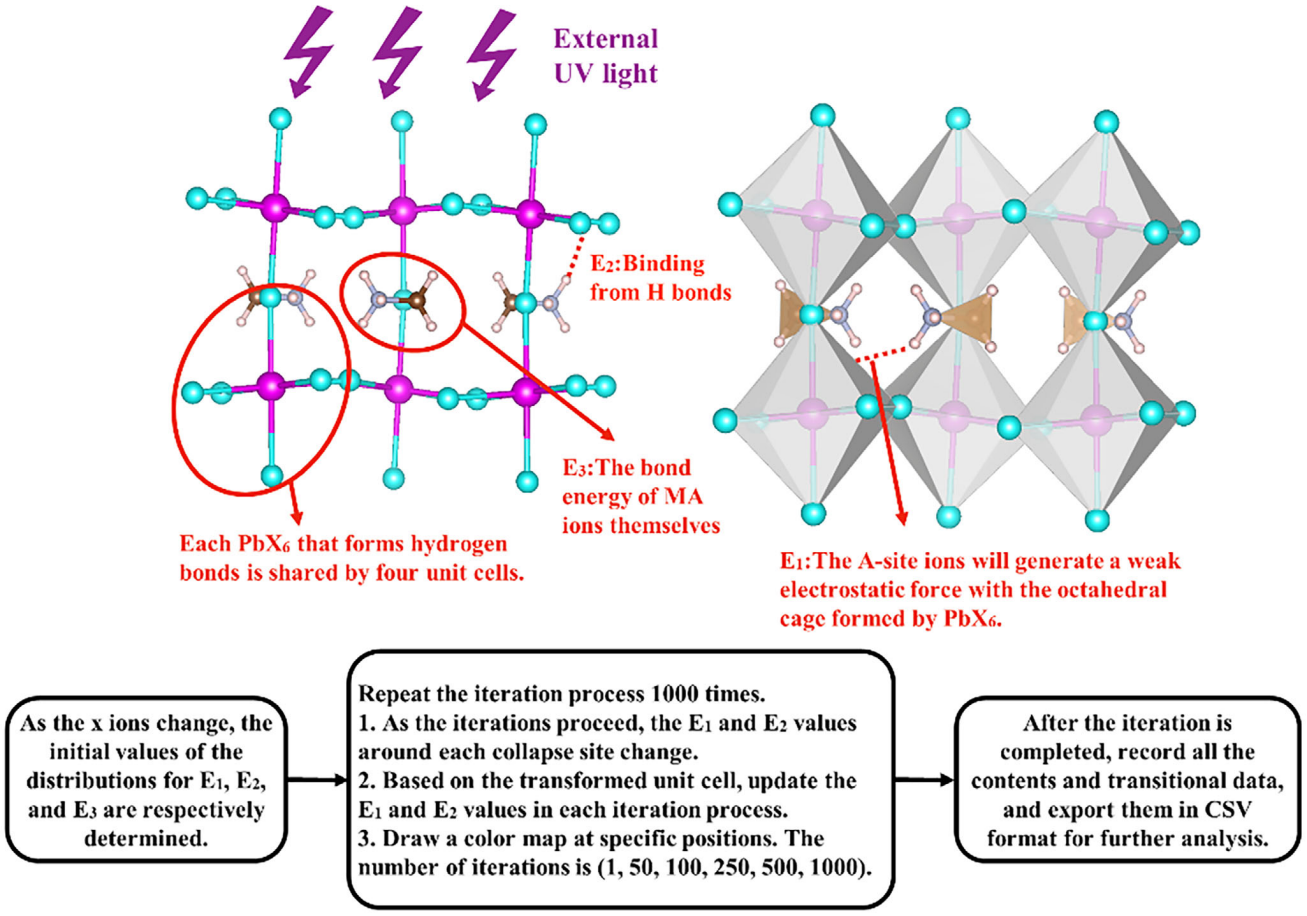
The modeling process of the perovskite unit cell.

Following our previous work,^[^ ^20^, ^21^, ^22^, ^23^, ^24^ ^]^ a 100 × 100 lattice was constructed for simulation, where each site represents the smallest repeating unit of a perovskite structure (**Figure** **1**). Based on the experiment results,^[^ ^25^, ^26^ ^]^ the degradation was initiated from the surface.

Each lattice was assigned several energy terms, including bonding energy B, degradation driving force D, and UV energy U. The overall activation energy of degradation is thus given by B‐D‐U, and the probability of structural breakdown P can be given by the classical kinetics calculation, which is the exponential of the overall activation energy divided by the universal gas constant and temperature.^[^ ^27^, ^28^ ^]^ According to the established thermodynamics and kinetics, the program node‐wise calculates the degradation probability (P) and diffusion direction (W) for each lattice point. Through cyclic iterations, the entire 2D lattice is updated continuously, achieving a dynamic simulation of the degradation process.

As each unit cell of the perovskite shows variable stability, due to the presence of imperfections and possible residual strains,^[^ ^29^, ^30^, ^31^, ^32^ ^]^ the initial B value of each simulation lattice was therefore randomly assigned between 0.59 and 0.98 eV to reflect the variance of the stability. Similarly, the initial D value was also randomly assigned between 0.58 and 0.88 eV for MAPbI_1−x_Br_x_, 0.23 and 0.45 eV for MAPbI_1−x_Cl_x_, and 0.36 and 0.53 eV for MAPbI_3_ to mirror the influence of the imperfections on each lattice.^[^ ^33^, ^34^ ^]^ Based on previous literature, the UV wavelength varies between 100 and 400 nm from Earth to space, with its intensity changes between 130 and 264 mW m^−2^. For the purpose of evaluating the influence of the UV light on the perovskite bonding, which is usually lowered by the radiation, with stronger intensity, reduces more of the bonding energy, the corresponding bonding energy reduction ranges from 0.1 to 0.49 eV.^[^ ^35^, ^36^, ^37^ ^]^ While the thermal effect showed much less impact on the energy terms, its influence on the stability is negligible compared to the high energy of UV light. Thus, U was set to be 0 eV under darkness and was gradually enhanced from 0.1 to 0.49 eV to simulate the near‐earth to near‐space UV intensity.^[^ ^38^, ^39^ ^]^ The overall activation energy of degradation is thus 0.09–0.39 eV for MAPbI_1−x_Br_x_.

Similarly, the doping ratio of Cl ions was set to be 43%, representing the formula of MAPbI_1.7_Cl_1.3_, which is among the most commonly reported formulations in previous literature.^[^ ^40^, ^41^ ^]^ Moreover, to illustrate the influence of the self‐assembled molecule additives, such as TTB/Zn‐TTB, on the degradation kinetics, simulations were also conducted by adding extra bonding energy of 0.1–0.2 eV,^[^ ^42^ ^]^ as the additives could form coordinate bonds with the perovskite.^[^ ^43^ ^]^ As the specific choice of additive does not significantly affect the simulation results, it is only necessary to simulate one of the most commonly applied additives, such as Zn‐TTB.

To accommodate the unconventional diffusion, as the host lattice was constantly degraded during the simulation, the connections are now assigned as variables (W) and will be updated with P. Each lattice point, which is associated with a probability of breakdown, P(x, y), has been connected by the probable paths to its left, right, upper, and down, represented by W (x, y, d = 1–4), whose initial value was set to be 0 at all lattices expect the first column in the left, which was set to be 1, as the oxygen and UV light attack the perovskite from the surface, which is represented by the 1st left column in the 100 × 100 lattices.

The update of the P value after each iteration was achieved by Eq. (1), which depicted the accelerated breakdown of the perovskite due to the host lattice having been changed due to the structural breakdown, as has been experimentally observed.

(1) $$\frac{dP_{i}}{dt}=\sum_{j}\left(P_{j}W_{ji}-P_{i}W_{ij}\right)\;$$

where W(x, y, d) represents the transition probability between a lattice site and its adjacent sites (left, right, up, and down). These W values are updated during the simulation. For instance, when P(x, y) reaches a certain threshold, the corresponding W value is changed to 1 to simulate decomposition or collapse. Based on our simulation rule, the change of the perovskite lattice after each iteration dP, is given by the right part of Eq. (1) times dt, whose value needs to be manually determined. If the value is set to be too large, the simulation will be divergent; on the other hand, if the value is too small, the simulation will take a long time to finish, resulting in unnecessary waste of time. To correlate dt with the real timescale, the following equation can be used:

(2) $$\tau_{\mathrm{hop}}=a^{2}/2nD$$

where D is the diffusion coefficient, τ_hop_ is the jump time per step, n is a constant related to the dimensions, and a is the lattice hopping distance of ion migration. According to the modeling and previous literatures,^[^ ^42^, ^43^, ^44^ ^]^ *n*  =  2*, D*  =  3.1 × 10^−9^ cm^2^/s and *a*  =  0.6 nm for iodine ion diffusion. The resulting timescale per step is approximately 2.9 × 10^−7^s, which reflects the physical timescale corresponding to one iteration step in the simulation.

If the value of dt is too small, the simulation will take too much time to finish; on the other hand, if the value of dt is too large, the simulation will not converge. For this reason, one could easily relate the stability enhancement by comparing the iteration numbers when the perovskite degrades completely.

For the simulation of UV light‐induced degradation, each lattice is initialized with a specific W value to initiate the process. As degradation proceeds, the values of W(x, y, d) are updated: once any P(x, y) exceeds 0.3, the W(left) or W(right) becomes 1.0 with a 50/50 probability, indicating complete degradation. When the value reaches 0.5, W(right) or W(left) also becomes 1.0, while W(up) and W(down) remain unchanged.

## 3 Results and Discussion

**Figure 2:**
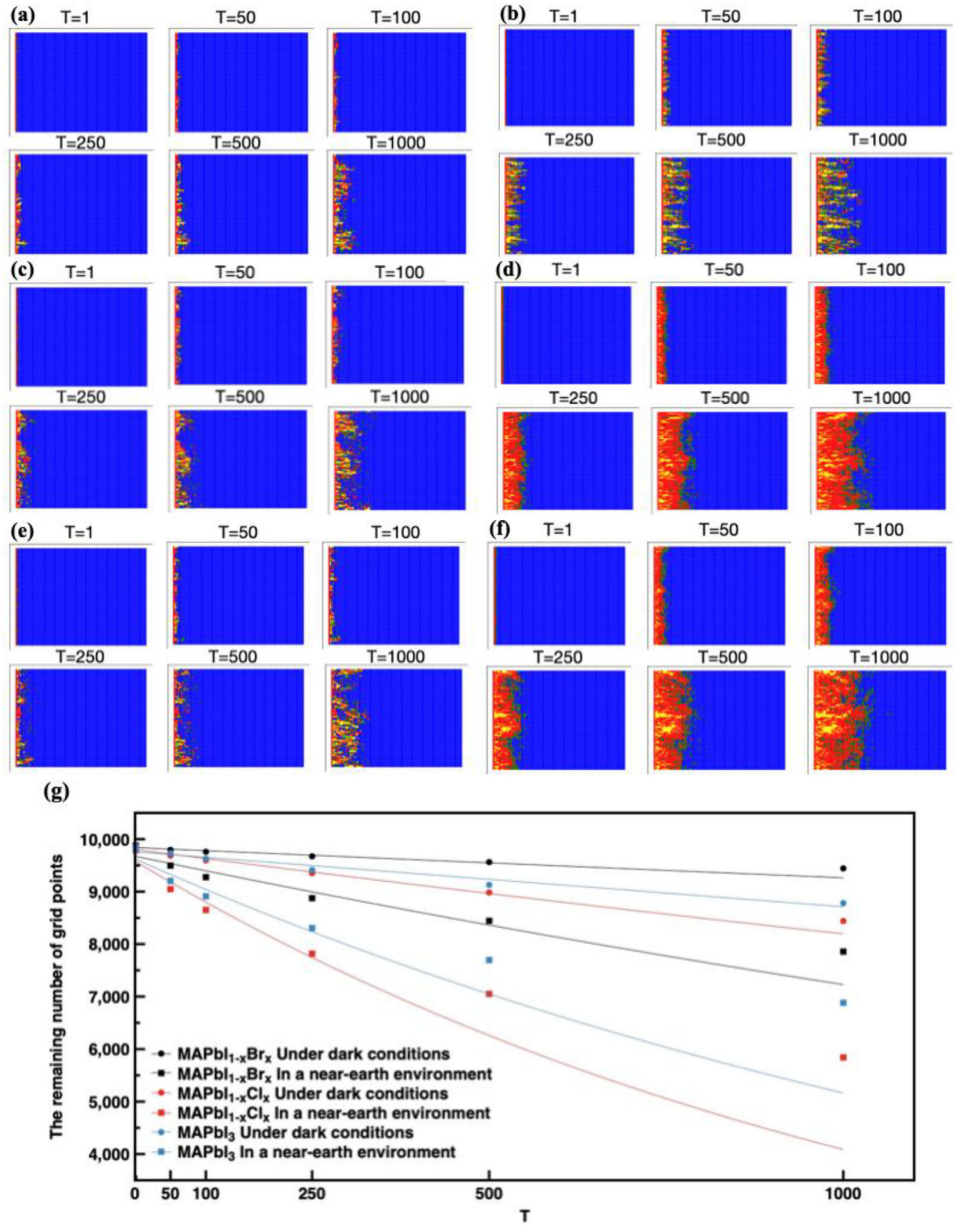
(a,c,e) are simulated results of MAPbI_1‐x_Br_x_, MAPbI_3,_ and MAPbI_1‐x_Cl_x_ in the dark environment, respectively. (b,d,f) are simulated results of MAPbI_1‐x_Br_x_, MAPbI_3_ and MAPbI_1‐x_Cl_x_ after ultraviolet irradiation in near‐earth, respectively; Blue = *p*: 0–0.3; green = *p*: 0.3–0.5; red = *p*: 0.5–1; and yellow = *p*: >1.0, iteration number represents the migration time. (g) is the plot of the remaining blue lattice as a function of the iteration numbers. The lines are the exponential decay curve fit, from top to bottom, the fitting curve functions are*f* (*x*) =  9840.33  × exp(− 6.04 × 10^−5^ *x*), *f* (*x*) =  9762.39 × exp(− 1.1 × 10^−4^ *x*), *f* (*x*) =  9803.31 × exp(− 1.8 × 10^−4^ *x*), *f* (*x*) =  9675.17 × exp(− 2.9 × 10^−4^ *x*), *f* (*x*) =  9623.09 × *e*xp(− 6.2 × 10^−4^ *x*) and *f* (*x*) =  9581.86 × exp(− 8.5 × 10^−4^ *x*). As can be observed from our fitted curve of the lattice collapse behavior, the curve demonstrates an exponential decay trend, where the degradation rate is initially rapid yet progressively slows before stabilizing. Such a phenomenon suggests a diffusion‐limited degradation mechanism, with no emergence of new degradation pathways or phase transition mechanisms. Therefore, the degradation behavior is unlikely to change even in extended iterations.

To investigate the influence of UV irradiation on perovskite materials, we simulated the lattice degradation process of three representative hybrid perovskites—MAPbI_1−x_Br_x_, MAPbI_3_, and MAPbI_1−x_Cl_x_. As the overall timescale of the simulation varies with the computing power, changing from a few seconds to hours, we have used iteration numbers for comparison, which gives a more straightforward interpretation of the stability enhancement. As can be seen in **Figure** **2**a,c,e, all the perovskites showed a limited amount of degradation in the dark environment, indicating that the destructive effect of water and oxygen is relatively slow in the absence of light. The MAPbI_1−x_Br_x_ showed the least amount of degraded lattice during the whole simulation (Figure 2a), as the stability is enhanced by Br ions, which enhanced the tolerance factor to 0.9, forming a stable cubic structure. Moreover, Br forms extra halogen bonds, which support the perovskite structure. As shown in Figure 2b,d,f, at the 1000th iteration under UV irradiation conditions, compared with the dark condition, the number of remaining perovskite lattice sites of Br‐doped perovskite decreased from 9443 to 7857, that of pure I perovskite decreased from 8780 to 6881, and that of Cl‐doped perovskite decreased from 8440 to 5840, respectively. In contrast, the MAPbI_3_ and MAPbI_1−x_Cl_x_ perovskites exhibited higher levels of degradation, as evidenced by Figure 2c–f, where the trend of degradation was plotted under both dark and UV light conditions for the three perovskites (Figure 2g). An exponential decay can be observed in the fitted line, following exactly the prediction of the kinetics.^[^ ^44^ ^]^

**Figure 3:**
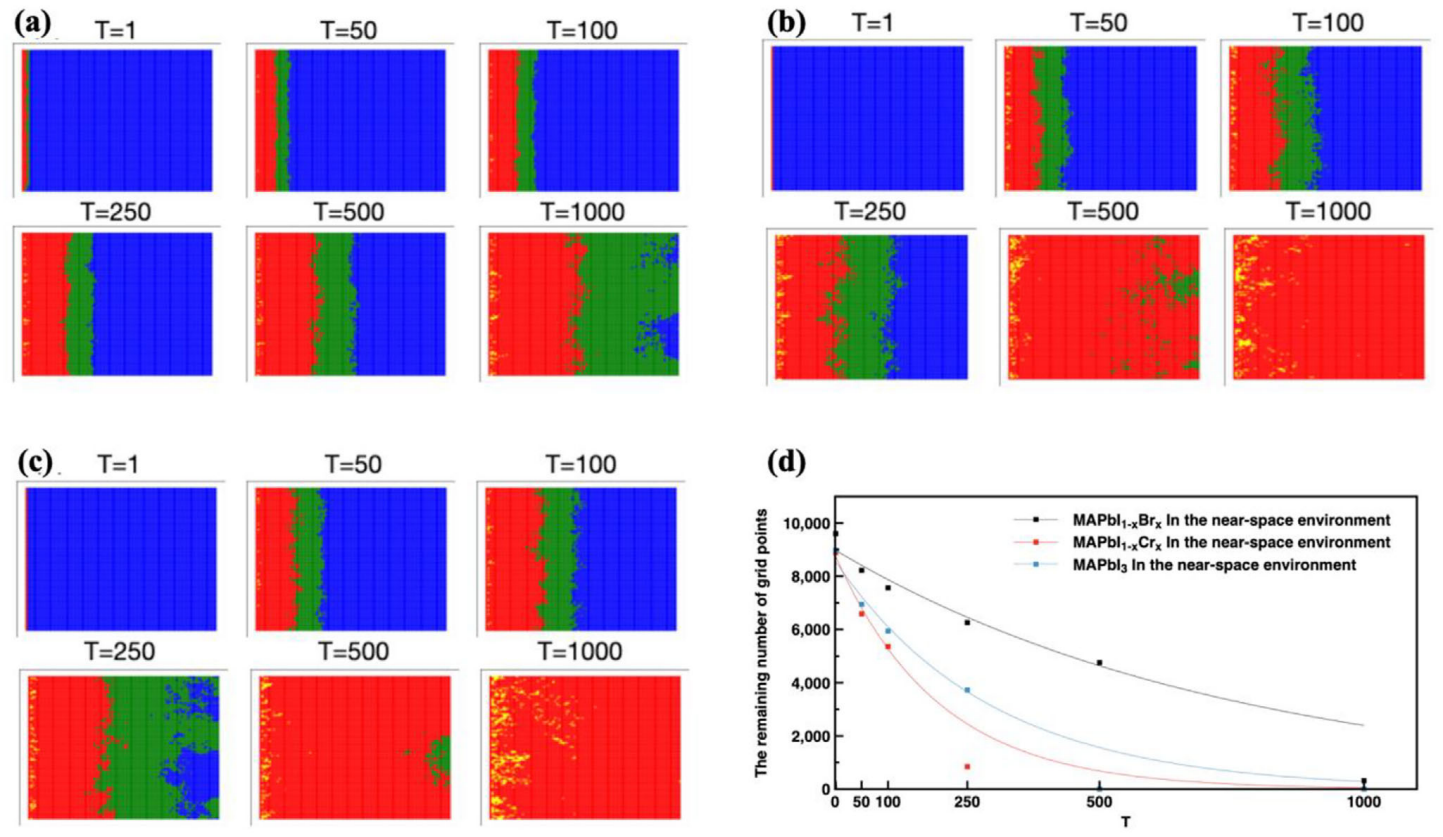
(a,b,c) are simulated results of MAPbI_1‐x_Br_x_, MAPbI_3,_ and MAPbI_1‐x_Cl_x_ after ultraviolet irradiation in near‐space, respectively; Blue = *p*: 0–0.3; green = *p*: 0.3–0.5; red = *p*: 0.5–1; and yellow = *p*: >1.0. (d) is the plot of the remaining blue lattice under near‐space ultraviolet irradiation as a function of the iteration numbers. The lines are the exponential decay curve fit, from top to bottom, the fitting curve functions are *f* (*x*) =  8986.21 × exp(− 1.3 × 10^−3^ *x*), *f* (*x*) =  8578.48 × exp(− 3.4 × 10^−3^ *x*), and *f* (*x*) =  8793.39 × exp(− 5.1 × 10^−3^ *x*).

To expand the understanding of the degradation process under the influence of the near‐space UV irradiation, which has much higher energy than normal UV light, further simulations were conducted (**Figure** **3**). It has been observed that all three perovskites showed much faster degradation rates, especially for MAPbI_3_, and MAPbI_1−x_Cl_x_, which were completely decomposed after 500 iterations (Figure 3b,c), while MAPbI_1−x_Br_x_ remained almost half of the original perovskite lattice (Figure 3a), demonstrating the importance of the Br bonding on the structural stability. In the near‐space environment, it can be found that the green grid is located to the right of the red grid and gradually extends to the right, which is inconsistent with the near‐earth and dark conditions. It might be a second‐order effect caused by the destruction of the area by high‐energy ultraviolet rays. The areas severely damaged by high‐intensity UV rays promoted the subsequent collapse of the perovskite lattice. It indicates that the relevant details of perovskite destruction in the near‐space environment still need further exploration and research.

**Figure 4:**
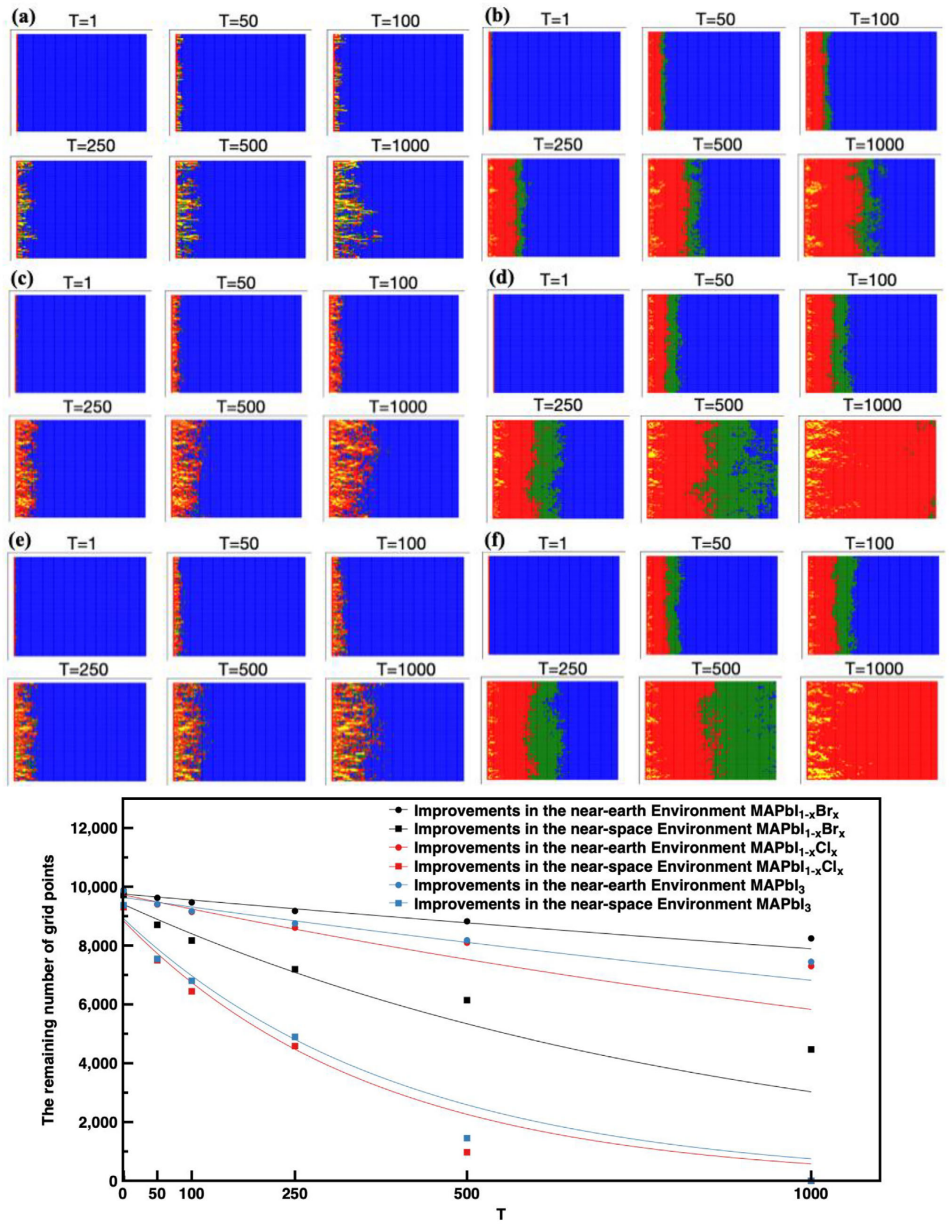
(a,c,e) are simulated results of MAPbI_1‐x_Br_x_, MAPbI_3_, and MAPbI_1‐x_Cl_x_ under near‐earth ultraviolet irradiation after the additive enhanced performance; (b,d,f) are simulated results of MAPbI_1‐x_Br_x_, MAPbI_3_ and MAPbI_1‐x_Cl_x_ under near‐space ultraviolet irradiation after the additive enhanced performance Blue = *p*: 0–0.3; green = *p*: 0.3–0.5; red = *p*: 0.5–1; and yellow = *p*: >1.0. (g) is the plot of the remaining blue lattice after adding the additive as a function of the iteration numbers. The lines are the exponential decay curve fit, from top to bottom, the fitting curve functions are *f* (*x*) =  9752.78 × exp(− 2.1 × 10^−4^ *x*), *f* (*x*) =  9638.47 × exp(− 3.4 × 10^−4^ *x*), *f* (*x*) =  9726.57 × exp(− 5.1 × 10^−4^ *x*), *f* (*x*) =  9408.38 × exp(− 1.1 × 10^−3^ *x*), *f* (*x*) =  8932.38 × exp(− 2.5 × 10^−3^ *x*), and *f* (*x*) =  8859.69 × exp(− 2.7 × 10^−3^ *x*).

To illustrate the influence of the additive, such as Zn‐TTB, extra bonding energy has been introduced, as TTB is able to form a coordinate bond with Pb. The decomposition process of all three perovskites under normal UV light and near‐space UV irradiation has been simulated. The degradation rate is obviously reduced after the application of the additive, as can be shown in **Figure** **4**a–f, not only because Zn‐TTB could form extra coordinate bonds with Pb in perovskite, which provided additional support for the stability, but also attributed to the formation of a passivating layer which hindered the ion migration, as has been previously observed by XPS, SEM, FTIR, and PL.^[^ ^31^ ^]^ During the simulation, extra bonding energy was introduced to simulate the influence of Zn‐TTB. Under near‐space ultraviolet irradiation, the number of remaining improved perovskite lattice sites at the 1000th iteration decreased from 8243 to 4470 for Br‐doped perovskite, from 7446 to 0 for pure I‐based perovskite, and from 7299 to 0 for Cl‐doped perovskite. Compared with near‐earth conditions, all three perovskite compositions exhibited accelerated degradation in the near‐space environment.

**Figure 5:**
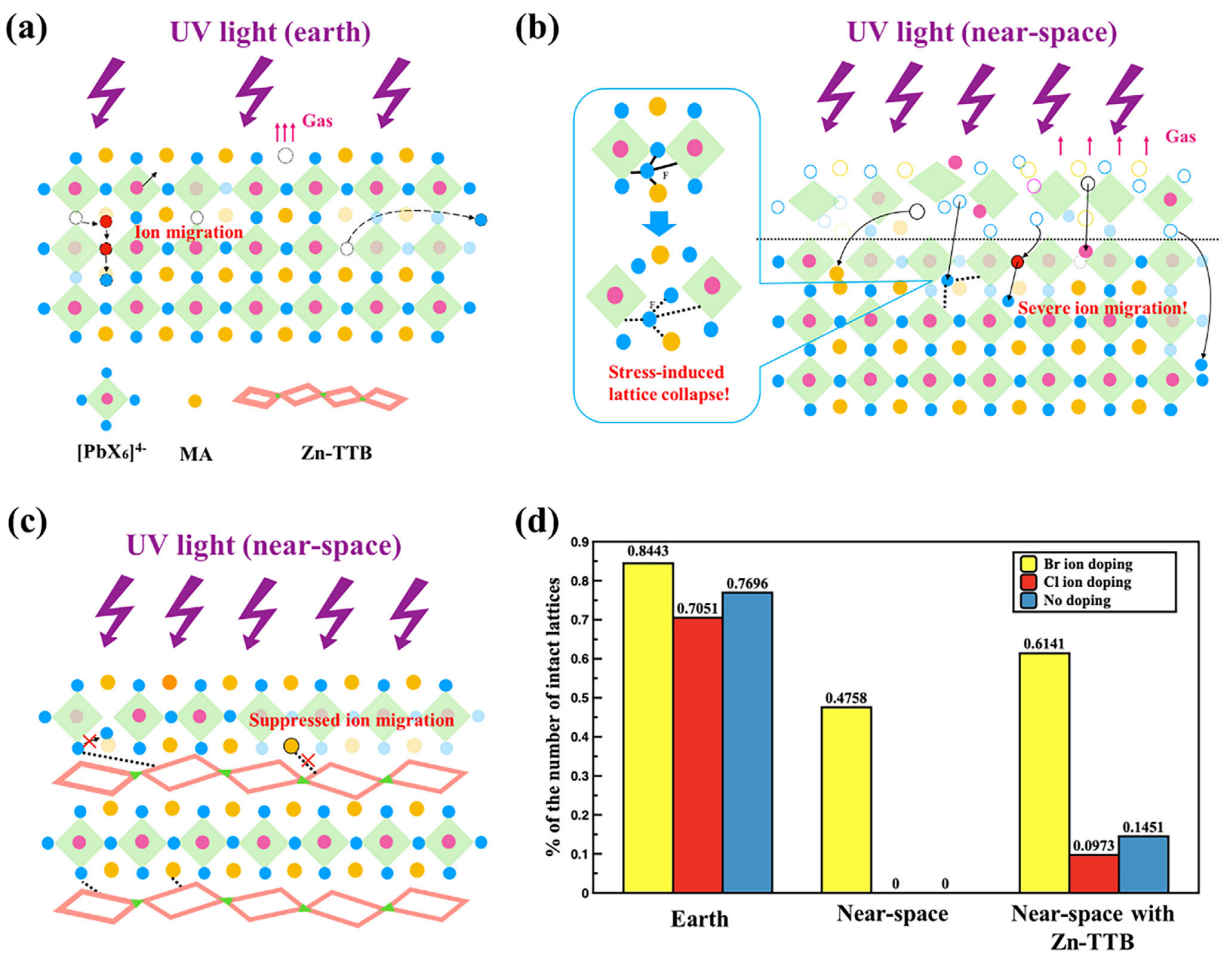
Mechanism of perovskite UV degradation: a) under earth, b) near‐space, and c) with Zn‐TTB additives. d) The remaining original perovskite lattice number under the three conditions at T = 500 (data were obtained from simulations).

Based on our simulation results and previous experimental observations,^[^ ^31^ ^]^ possible damaging mechanisms of the perovskite lattice by UV light in earth (**Figure** **5**a) and near‐space (Figure 5b) environments can be extrapolated. The degradation was initiated by the ion migration and decomposition of organic cations into volatile gases,^[^ ^45^, ^46^ ^]^ which is accelerated by the UV light, as evidenced by Figures 3 and 4. The degradation rate can be further enhanced under near‐space conditions, obviously due to the higher UV intensity. The application of additives, such as Zn‐TTB, is capable of providing extra bonding energy,^[^ ^47^, ^48^ ^]^ as shown in Figure 5c, which successfully reduced the degradation rate.

## 4 Conclusion

To conclude, this study revealed the detailed kinetics of how UV irradiation affects hybrid perovskite materials by simulating the degradation process of MAPbI_1−x_Br_x_, MAPbI_3_, and MAPbI_1−x_Cl_x_. Under dark conditions, all three perovskites degrade slowly, which is accelerated by the UV exposure, particularly for MAPbI_3_ and MAPbI_1−x_Cl_x_, due possibly to the presence of defects in the perovskite. The degradation rate was successfully reduced in the following simulation, where the defects were passivated by the addition of Zn‐TTB, which significantly hindered degradation in all simulated perovskites, highlighting the potential of additives to enhance structural stability and extend the lifespan of these materials under UV exposure. Such a strategy is also expected to be employed in large‐area perovskite solar modules. However, the possible effects of Zn‐TTB on perovskite crystallization via distinct methods (e.g., blade coating, slot‐die coating, etc.) need to be elucidated. Hopefully, these discoveries will not only enhance the scientific understanding of the perovskite degradation under irradiation but also pave the way for practical applications.

## Conflict of Interest

The authors declare no conflict of interest.

## Data Availability

The data that support the findings of this study are available from the corresponding author upon reasonable request.
